# Diffusion of MMPs on the Surface of Collagen Fibrils: The Mobile Cell Surface – Collagen Substratum Interface

**DOI:** 10.1371/journal.pone.0024029

**Published:** 2011-09-01

**Authors:** Ivan E. Collier, Wesley Legant, Barry Marmer, Olga Lubman, Saveez Saffarian, Tetsuro Wakatsuki, Elliot Elson, Gregory I. Goldberg

**Affiliations:** 1 Division of Dermatology, Department of Medicine, Washington University School of Medicine, Saint Louis, Missouri, United States of America; 2 Department of Biochemistry and Molecular Biophysics, Washington University School of Medicine, Saint Louis, Missouri, United States of America; 3 Department of Pathology, Washington University School of Medicine, Saint Louis, Missouri, United States of America; 4 Department of Bioengineering, University of Pennsylvania, Philadelphia, Pennsylvania, United States of America; 5 Department of Physics, University of Utah, Salt Lake City, Utah, United States of America; 6 Department of Physiology, Biotechnology and Bioengineering Center, Medical College of Wisconsin, Milwaukee, Wisconsin, United States of America; Massachusetts Institute of Technology, United States of America

## Abstract

Remodeling of the extracellular matrix catalyzed by MMPs is central to morphogenetic phenomena during development and wound healing as well as in numerous pathologic conditions such as fibrosis and cancer. We have previously demonstrated that secreted MMP-2 is tethered to the cell surface and activated by MT1-MMP/TIMP-2-dependent mechanism. The resulting cell-surface collagenolytic complex (MT1-MMP)_2_/TIMP-2/MMP-2 can initiate (MT1-MMP) and complete (MMP-2) degradation of an underlying collagen fibril. The following question remained: What is the mechanism of substrate recognition involving the two structures of relatively restricted mobility, the cell surface enzymatic complex and a collagen fibril embedded in the ECM? Here we demonstrate that all the components of the complex are capable of processive movement on a surface of the collagen fibril. The mechanism of MT1-MMP movement is a biased diffusion with the bias component dependent on the proteolysis of its substrate, not adenosine triphosphate (ATP) hydrolysis. It is similar to that of the MMP-1 Brownian ratchet we described earlier. In addition, both MMP-2 and MMP-9 as well as their respective complexes with TIMP-1 and -2 are capable of Brownian diffusion on the surface of native collagen fibrils without noticeable dissociation while the dimerization of MMP-9 renders the enzyme immobile. Most instructive is the finding that the inactivation of the enzymatic activity of MT1-MMP has a detectable negative effect on the cell force developed in miniaturized 3D tissue constructs. We propose that the collagenolytic complex (MT1-MMP)_2_/TIMP-2/MMP-2 represents a Mobile Cell Surface – Collagen Substratum Interface. The biological implications of MT1-MMP acting as a molecular ratchet tethered to the cell surface in complex with MMP-2 suggest a new mechanism for the role of spatially regulated peri-cellular proteolysis in cell-matrix interactions.

## Introduction

The three-dimensional scaffold of vertebrate extracellular matrix (ECM) is a highly organized, insoluble assembly of large protein molecules, including collagens, proteoglycans, fibronectin, laminin, as well as others. These proteins give tensile strength to the tissue, but also act to limit the mobility of constituent cells [1], [2]. To cope with these restrictions during normal morphogenetic events, or during pathologic remodeling, resident cells of tissues can secrete a specialized group of enzymes, matrix metalloproteases (MMPs) [3]–[5], that can degrade ECM macromolecules such as collagens and proteoglycans. The role of MMPs in both normal and pathological processes characterized by intensified tissue remodeling has been recognized for a long time. Numerous studies in vitro and in animal models pointed to the importance of these enzymes in metastatic invasion of tumor cells into the surrounding connective tissue [3]–[5]. The large number of distinct MMPs with relatively broad and often overlapping substrate specificities as well as the increasing number of protein substrates made the goal of assigning a particular biological function to a specific MMP difficult. Over the last few years this picture has seen a considerable improvement largely due to the development of mouse models including one with a mutated collagenase cleavage site and others that ablate the genes of several MMPs and their inhibitors [6]–[9].

Understanding of the mechanisms of spatial control of MMP-catalyzed extracellular proteolysis has also significantly progressed. These mechanisms involve cell surface tethered MMPs [10], [11], binding of soluble MMPs to the cell surface and in situ activation [10]; and, recently, a diffusion based mode of interaction of the enzymes with the underling ECM substrata [12]–[15], all contributing to the sequestering of the enzymatic activity to the specific structures of cell – ECM interface. Our recent results including those presented here, establish that MMP-1, -2, -9 and MT1-MMP can diffuse laterally on the collagen substrate surface without noticeable dissociation. Most interestingly, we have shown that activated MMP-1 is a novel type of diffusion-based, “Burnt Bridge” Brownian Ratchet capable of biased diffusion on the surface of collagen fibrils [13], [14]. The bias portion of MMP-1 diffusion is driven by proteolysis of its substrate, collagen, and not ATP hydrolysis [16] The lateral diffusion of MMP-2 requires the hemopexin-like C-terminal domain. Complex formation of pro-MMP-2 and -9 with inhibitors TIMP-2 and -1, respectively, does not interfere with the diffusion process, in spite of the fact that these inhibitors occupy a significant portion of the solvent exposed surface of the C-terminal domain. This finding implies that the cell surface activation complex of MT1-MMP/TIMP-2/MMP-2 is mobile relative to the underlying collagen substratum. We propose a model of the dynamic cell surface – collagen substratum interface that is of considerable interest for mechanistic understanding of the dynamic cell – ECM interactions and the role of proteolysis catalyzed by MMPs in controlled cell spreading and motility.

## Results

### The Extracellular Moiety of the Trans-Membrane Protease MT1-MMP is a Proteolysis Driven Brownian Ratchet Capable of Biased Diffusion on The Surface of Collagen Fibrils

Activated trans-membrane protease MT1-MMP acts as a cell surface receptor for tissue inhibitor of metalloprotease 2 (TIMP-2) with Kd = 2.54×10^−9^ M. The MT1-MMP/TIMP-2 complex in turn acts as a receptor for the soluble MMP-2 (Kd = 0.56×10^−9^ M), binding the TIMP-2 to the carboxyl-end domain of the enzyme [10], [17]. Recently, evidence has accumulated that MT1-MMP dimerizes on the cell surface to produce a (MT1-MMP)_2_/TIMP-2/MMP-2 complex and the activated MT1-MMP can initiate the digestion of native collagen fibrils similar to the secreted collagenase, MMP-1 [11], [18]–[22]. However, the mechanism of substrate recognition between the two structures of relatively restricted mobility, the cell surface collagenolytic complex and the ECM embedded collagen fibril, remained unclear.

**Figure 1:**
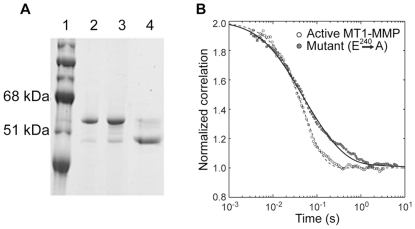
Biased Diffusion exhibited by MT1-MMP on The Surface of Collagen Fibrils is absent in a catalytically inactive mutant E240->A. (**A**). SDS-PAGE analysis of Gel filtration Chromatography of the MT1-MMP expressed in E.Coli: MW standards (lane 1); the leading portion of the major protein peak (lane 2); trailing portion of the major protein peak (lane 3); activation of the MT1-MMP (lane 3) with recombinant Furin for 30 min at 300C (lane 4). (B). Normalized experimental correlation functions obtained from collagen fibrils decorated with either activated MT1-MMP Wild Type (open circles) or MT1-MMP inactive mutant (E240->A, closed circles) were calculated from the 400 µs binned data records as described in Methods. The background was suppressed using the spatial background filter described earlier [13], see methods). Three experimental correlation functions for each enzyme were normalized and then averaged to obtain the data shown. The experimental correlation function for wild-type enzyme was fitted (Methods, equation 3) to a 1D diffusion (D = 6.0±0.05×10^−9^ cm^2^ s^−1^) plus flow (V = 5.8±0.2 µm s^−1^) model. The correlation function of the inactive mutant exhibits a long tail characteristic of an unbiased 1-D diffusion, accordingly, a fit of the same equation (Methods, equation 3) yielded a local diffusion coefficient (D = 1.1±0.04×10^−8^ cm^2^ s^−1^) and no significant flow.

Here we examine whether the extracellular portion of the membrane collagenase, MT1-MMP, containing the catalytic and the hemopexin-like domains is capable of diffusion on collagen fibrils and whether its collagenolytic activity translates into directional motion as in the case of MMP-1. We expressed the extracellular portion of wild type and inactive mutant MT1-MMP pro-enzyme forms lacking trans-membrane and cytoplasmic domains. The inactive mutant of MT1-MMP contained a single amino acid residue substitution (E^240^->A) in the active center of the enzyme. (Figure 1A, see methods). We labeled the enzymes with the fluorescent dye Alexa^488^, and used those to decorate oriented collagen gels polymerized from acid-soluble rat-tail collagen in the presence of magnetic field of 8T. The motion of the enzymes on collagen fibrils was monitored using two-photon excitation correlation spectroscopy (FCS) [14]. We found that the experimental correlation function for the active enzyme (Fig. 1B) fit well to a 1D diffusion plus flow model [23]. For 1D diffusion the correlation function has an elongated tail at longer times as shown by the inactive MT1-MMP mutant. In contrast the correlation function for active MT1-MMP lacks this tail due to the presence of directional flow (Fig. 1B) [23], [24]. The local diffusion coefficient for the active enzyme, D = 0.6±0.05×10^−8^ cm^2^ s^−1^ and the transport velocity V = 5.8±0.2 µm s^−1^, were determined from the fit of the correlation function obtained from the wild-type activated MT1-MMP. The diffusion coefficient for the inactive mutant, D = 1.1±0.04×10^−8^ cm^2^ s^−1^, was similar to the diffusion coefficient of the active enzyme, which suggests that inactivation has little effect on the diffusion properties of MT1-MMP on the collagen fibrils and that the transport mechanism (biased diffusion) depends on the catalytic activity of the enzyme. The values of the diffusion coefficient obtained for wild-type and mutant MT1-MMP on collagen fibrils are in good agreement with those for MMP-1 on collagen fibrils that we have determined earlier [12].

How would the added frictional resistance of the membrane affect the rate of diffusion of MT1-MMP, an integral membrane protein, along the fibrils? Diffusion coefficients of membrane proteins range from ∼10^−8^ to ∼10^−10^ cm^2^ s^−1^ [25] corresponding to a 100 fold increase in the frictional resistance which is inversely proportional to the diffusion coefficient. The diffusion coefficient of MT1-MMP on a collagen fibril in aqueous medium is 0.6×10^−8^ cm^2^ s^−1^. If the membrane diffusion coefficient of MT1-MMP is at the higher end of the range, the effect of membrane attachment on fibrillar diffusion will be relatively minor because the frictional resistance to diffusion of the membrane and the fibril are of similar magnitude. However, the fibrillar diffusion of the embedded enzyme could be slowed by as much as ∼100-fold if the membrane diffusion coefficient is at the lower end of the range because this would correspond to a frictional resistance 100 times greater than that of the fibril. Further experiments are needed to understand the motion parameters of the enzyme on the cell surface.

**Figure 2:**
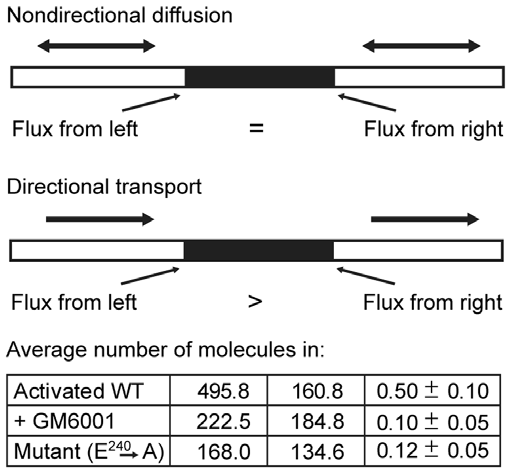
The unequal flux of MT1- MMP molecules around a no-transport block on the collagen fibril. Photobleaching of MT1-MMP decorated collagen fibrils with a laser beam intensity of 80 to 90 mW prevents recovery after photobleaching indicating damage to the fibril, thus blocking enzyme transport across the bleached area. The average number of single enzyme molecules passing through the laser beam in the left (CL) and right (CR) flanks of the no-transport block are calculated by counting (see Methods) and then averaging the number of spikes in the 300 s fluorescence records of five independent experiments for each form of the enzyme. The difference in transport at the flanks for each experiment is expressed as the asymmetry ratio, |(CL−CR)/(CL+CR)|, which is then averaged over all experiments. A ratio of 1 indicates perfect flow asymmetry and 0 indicates perfectly symmetric flow. The graphic portion is reproduced from [13]. License Number 2257830858271 issued Aug 28, 2009 by The American Association for the Advancement of Science.

To confirm the presence of directional flow evident in the FCS experiments and ascertain whether biased transport dominates the random diffusion over longer distances, we measured the flux of single MT1-MMP molecules on each side of a no-transport block, created by exposure of a collagen fibril to a laser beam of high power (80–90 mW). This procedure damages the fibril so that no recovery of fluorescence is observed indicating the absence of enzyme transport across the exposed area (Fig. 2). . If transport is directional, the flux of molecules, which have accumulated near the upstream border of the exposed area will be greater than the flux near the downstream border, which have been depleted by directional transport away from the border. Equal numbers of spikes at each border indicate random diffusion. To quantify the extent of directional transport, we define the asymmetry ratio as the number of spikes on the upstream edge minus the number on the downstream edge divided by the sum of two. An asymmetry ratio of 1 indicates perfect directional flow while a ratio of 0 indicates perfectly symmetric diffusion. Confirming our FCS results, we observed an asymmetry ratio (Fig. 2) of 0.5 for the activated MT1-MMP which indicates the occurrence of directional flow. In the presence of MMP inhibitor GM6001, the asymmetry ratio was reduced to 0.1, a value similar to that obtained for the inactive mutant. These results show that transport can dominate random diffusion over a considerable distance and is dependent on collagen proteolysis. These results prompted us to ask whether the remaining components of the cell surface collagenolytic complex (MT1-MMP)_2_/TIMP-2/MMP-2 are able to diffuse laterally on a collagen fibril and if so what are characteristics of their mobility?

### MMP-2 and -9 and their respective complexes with the inhibitors TIMP-2 and TIMP-1 are capable of random processive diffusion on the surface of Collagen Fibrils

Previously we have shown that the bulk of the MMP-2 binding to a gelatin layer depends on its ability to diffuse laterally on its surface [12]. The lateral diffusion is greatly facilitated by the C-terminal hemopexin-like domain of the enzyme whereas the specificity of binding resides with the fibronectin-like gelatin/collagen binding domain [26], [27]. Here we examine whether a closely related gelatinase, MMP-9, is capable of lateral diffusion on the surface of the artificial gelatin layer and whether either or both MMP-2 and -9 can diffuse on the surface of native collagen fibrils, a physiologically relevant component of ECM. In contrast to MMP-1, MMP-9 and MMP-2 pro-enzymes form specific complexes with TIMP-1 and the related inhibitor TIMP-2, respectively [28]. These complexes are formed via interaction between the inhibitors and hemopexin-like C-terminal domains of the enzymes [29], [30] which occupies a major portion of the solvent exposed surface of this domain [31]–[33]. Since the C-terminal domain of MMP-2 is required for diffusion on gelatin and its complex with TIMP-2 is an essential component of the cell surface enzyme activation complex, it is of special interest to determine the effect of the inhibitor binding on the diffusion properties of the enzymes.

**Figure 3:**
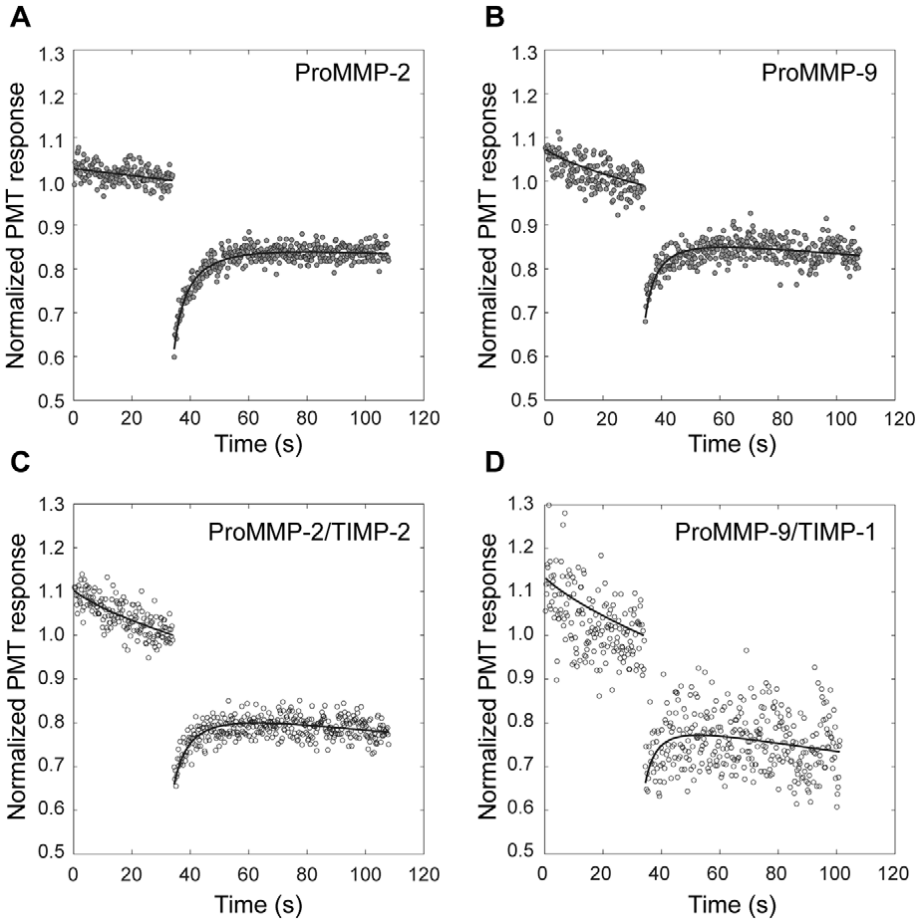
Fluorescence photobleaching recovery from gelatin-adsorbed fluorescently labeled MMP-2 (A), MMP-9 (B) and their complexes with inhibitors TIMP-2 and TIMP1 (C) and (D). Fluorescence photobleaching recovery (FPR) curves were obtained from fluorescently labeled enzymes adsorbed to gelatin coated glass coverslips. Fluorescence was excited by an attenuated Argon-ion laser (488 nm) beam (0.25±0.04 µWatt) focused on the decorated gelatin layer. It was monitored through a 40× microscope objective with a photomultiplier tube (PMT) [92] for 36 s before and 72 s after the delivery of a 50 ms bleaching pulse (187±19 µWatt). The fluorescence is normalized to the mean value of the fluorescence signal (PMT response) just prior to the delivery of the bleaching pulse. The solid lines are a non-linear least squares fit of the predicted diffusion dependent recovery ([12], Eq 3.2) to the fluorescence data. The local diffusion coefficients obtained from fitting are summarized in Table 1.

We used fluorescence photobleaching recovery (FPR) to test the ability of purified Alexa 488™ fluorescently labeled enzymes to diffuse on gelatin layers as described previously [12]. The isolated recovery phase of the entire FPR experiment is shown in Fig. 3 A&C (for MMP-2 and MMP-2/TIMP-2 complex) and Fig. 3 B&D (for MMP-9 and MMP-9/TIMP-1 complex). The signal has been normalized to the value of fluorescence just prior to photo-bleaching. The recovery of pro-MMP-2 (Fig. 3A) and pro-MMP-2/TIMP-2 complex (Fig. 3C) indicates diffusive motion of the surface bound ligands. The solid lines in both figures are determined by non-linear least squares fit of the data to the predicted diffusion-dependent recovery equation as described previously [12]. The diffusion coefficients of 2.4×10^−9^ and 1.7×10^−9^ cm^2^ s^−1^, and the mobile fractions of 0.7 and 0.6 were obtained for MMP-2 and MMP-2/TIMP-2 complex respectively. Table 1 shows the mean diffusion coefficients as well as their standard deviations obtained in these experiments. The average diffusion coefficient of MMP-2/TIMP-2 complex is 37% smaller than that of the MMP-2 suggesting only a slight inhibition of the mobility by the complex formation with TIMP-2. The diffusion-dependent recovery of MMP-9 (Figure 3B) with the average diffusion coefficient of 2.5×10^−9^ cm^2^ s^−1^ (Table 1) and mobile fraction of 0.7 does not differ significantly from that of MMP-2. The recovery of MMP-9/TIMP-1 complex seen in Figure 3D has a diffusion coefficient of 2.3×10^−9^ cm^2^ s^−1^ and mobile fraction of 0.5 showing that the rate of diffusion of MMP-9 is not affected by complex with TIMP-1, suggesting that the inhibitor is strategically positioned on the surface of the C-terminal domain not to interfere with the substrate interaction.

**Table 1:** Diffusion Coefficients and Transport Velocities of MMPs on Gelatin and Collagen Surfaces.

MMP mobility on Collagen Fibrils				MMP mobility on Gelatin
Protease	τ_D_ (ms)	D (cm^2^ s^−1^)×10^8^	V (µm s^−1^)	D (cm^2^ s^−1^)×10^8^
**MMP2**	17.5±0.7	1.29±0.05	n.d.	0.23±0.06
**MMP2/TIMP2 Complex**	12.4±0.7	1.8±0.1	n.d.	0.15±0.02
**MMP9**	38.1±1.4	0.6±0.02	n.d.	0.25±0.06
**MMP9/TIMP1 Complex**	18.1±1.4	1.2±0.1	n.d.	0.23
**MMP9 Homodimer**	Immobile on both substrates			
MMP1**	28±4.4	0.8±0.15	4.5±0.4	-
**MT1-MMP**	35±2.4	0.6±0.05	5.8±0.2	-
**MT1-MMP (E^240^->A) mutant**	20.6±0.8	1.1±0.04	n.d.	-

Both MMP-2 and -9 bind to native type I collagen fibrils in either pro- or activated enzyme form and, although both enzymes are very active against denatured collagen, gelatin, native fibrillar collagen is not a substrate for either of the enzymes [34]. We detect no digestion of collagen fibrils reconstituted from acid soluble rat tail collagen by activated MMP-2 or MMP-9 even after lengthy (48 hr) incubations, whereas similar concentrations (1–2 µg/50 µl gel) of MMP-1 solubilize such fibrils within 1 h (data not shown) raising the possibility that reported activity of MMP-2 against molecular [35]–[37] and fibrillar [36], [37] forms of collagen is due to the presence of partially denatured collagen in the reaction mix. Collagen monomers are stable at 37°C when packed into a fibril but in solution can unfold slowly with a concomitant loss of ellipticity [38]. Collagenolysis of the monomeric collagen catalyzed by MMP-2 may be due to an attack on partially unfolded monomers particularly where long incubations are involved. Thus a cooperative action of collagenases and gelatinases is required to complete the degradation of a collagen fibril.

**Figure 4:**
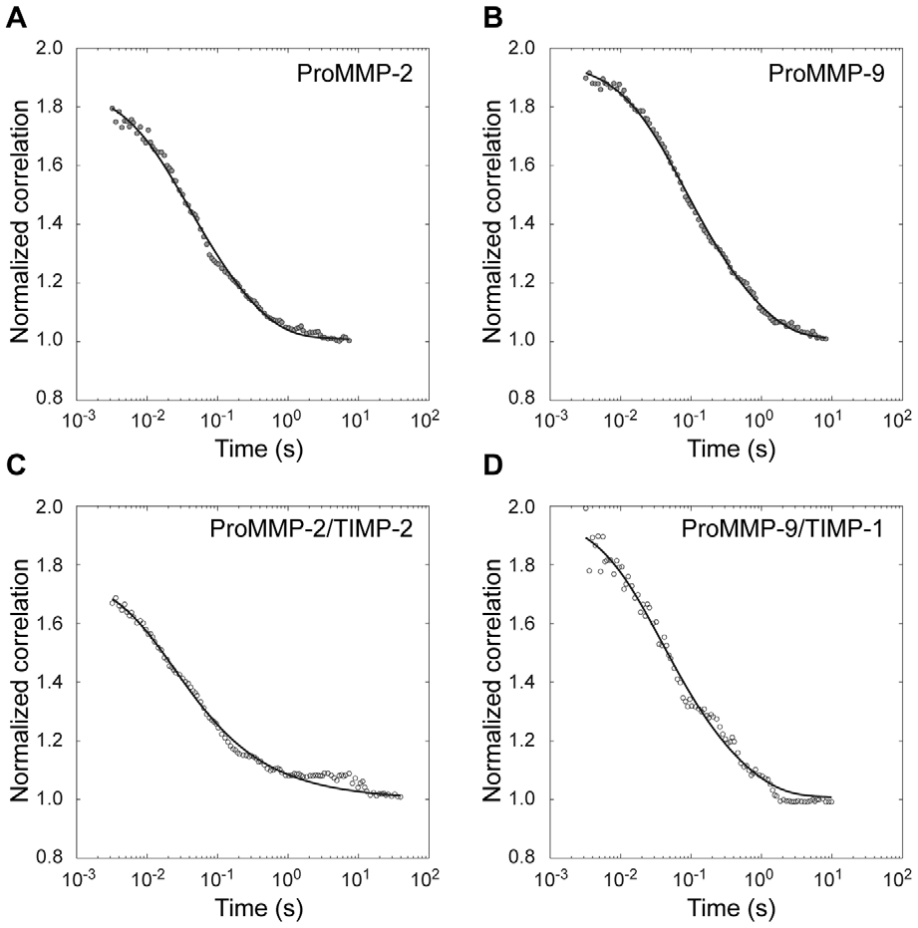
Fluorescence Correlation Spectroscopy of MMP-2, MMP-9 and their respective complexes with inhibitors TIMP-2 and TIMP-1 on collagen fibrils. Normalized experimental correlation functions obtained from collagen fibrils decorated with MMP-2 (A), MMP-2/TIMP-2 (C), MMP-9 (B) and MMP-9/TIMP-1 (D) were calculated as described in Fig. 1. The experimental correlation functions were fitted to an 1-D unbiased diffusion model (Methods, Eq. 2). The local diffusion coefficients obtained from the fit are summarized in Table 1.

We next examined whether MMP-2 and MMP -9 can diffuse on the surface of an individual collagen fibril, a physiologically relevant substrate as opposed to a gelatin layer. Again we used two-photon FCS to observe the interaction of MMP-2 and -9 molecules with individual collagen fibrils in a hydrated collagen gel as described above for MT1-MMP and earlier for MMP-1 [14]. The experimental correlation functions for the MMP-2 (Fig. 4A), MMP-2/TIMP-2 complex (Fig. 4B), MMP-9 (Fig. 4C) and MMP-9/TIMP-1 complex (Fig. 4D) fit well to a 1D free diffusion model [13] without the directional flow component. The local diffusion coefficients (Table 1) were determined from each of the normalized correlation functions. The estimated diffusion coefficients for MMP-2, MMP-9 and their respective TIMP complexes are significantly higher than that of the same ligands on gelatin (Table 1) and less than two times that of MMP-1 on the fibril substrate [13]. These findings indicate that MMP-2 and 9 and their respective complexes with inhibitors TIMP-2 and TIMP-1 are able to move processively on the surface of native collagen fibril. The movement is a random walk without a biased motion component in agreement with the fact that native collagen fibril is not digested by either of these two enzymes. These data show that each component and thus the entire cell-surface collagenolytic complex are mobile with respect to the substrate. Although it is attractive to hypothesize that this motion could potentially be dominated by the biased diffusion of the MT1-MMP dimer further experiments with assembled complex are necessary to ascertain this possibility.

### Dimerization of MMP-9 Occurs via Formation of an Intermolecular Disulfide Bridge Between Cysteine Residues Cys^478^ and Renders the Enzyme Immobile Both on Gelatin and Collagen Fibril surfaces

In addition to the complex with TIMP1, MMP-9 can form a covalent homodimer and a complex with MMP-1 as we have previously demonstrated [30]. The formation of the MMP-9 pro-enzyme complex with TIMP-1 prevents dimerization. Since complex formation of MMP-9 with TIMP-1 does not inhibit the diffusion of the enzyme it was of interest to examine the effect of dimerization on the rate of diffusion.

**Figure 5:**
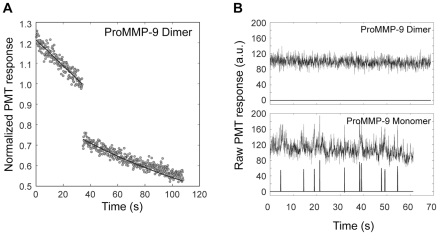
Dimerization of MMP-9 Renders the Enzyme Immobile Both on Gelatin Layers and Collagen Fibril surfaces. **A.** FPR curve for fluorescently labeled gelatin-adsorbed MMP-9 homodimer was obtained as described above in Figure 3. No detectable increase of fluorescence (open circles) occurs after the photobleaching pulse indicating the immobility of the MMP-9 homodimer. Monitoring beam photobleaching alone, indicated by the solid curves (Eq. 3.2 [12] with mobile fraction = 0), accounts for the decay of fluorescence before and after the photobleaching pulse. **B (upper panel)**. A primary fluorescence record, rebinned to 40 ms (upper curve), was obtained from an individual fibril decorated with fluorescently labeled MMP-9 homodimer. The background noise in the primary record was suppressed by applying the spatial background filter described earlier [13] to reveal a flat baseline (lower curve) indicating the absence of single molecule spikes. **B (lower panel)**. A primary fluoresence record, rebinned to 40 ms (upper curve), was obtained from an individual fibril decorated with fluorescently labeled MMP-9 monomer. The background noise in the primary record was suppressed by applying the same spatial background filter to reveal significant fluctuations in fluoresence (lower curve) indicating the presence of single molecule spikes.

An FPR experiment with purified fluorescently labeled proMMP-9 homodimer bound to gelatin showed no detectable recovery (Fig. 5A.) indicating that the gelatin bound enzyme is completely immobile. The mobility of the MMP-9 homodimer on the surface of collagen fibrils was examined in an FCS experiment. The absence of fluorescent spikes in the record from the fibril-bound MMP-9 homodimer (Figure 5B, upper panel) compared to the MMP-9 monomer record (Figure 5B, lower panel) indicates that the enzyme is immobile on the surface of the collagen fibril as it is on the gelatin layer. The complete inhibition of the diffusion by dimerization of MMP-9 raises the question of the mechanism of MMP-9 dimerization.

**Figure 6:**
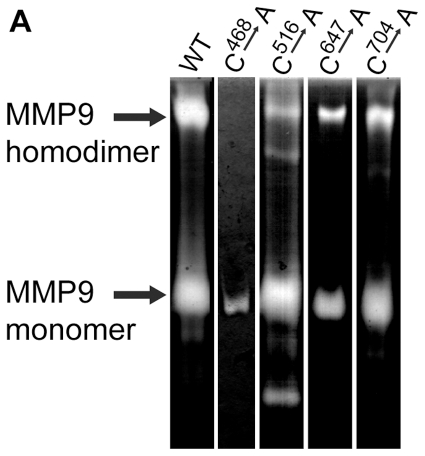
Zymogram analysis of MMP-9 Cysteine Replacement Mutants C^468^->A, C^516^->A, C^674^->A, C^704^->A. Individual cDNAs bearing the mutant substitutions as well as the wild type were expressed as described [30]. Samples of serum-free conditioned media (5–100 µl) were dialyzed, lyophilized, and subjected to NaDodS04-PAGE using a 7% acrylamide gel impregnated with gelatin. A zymogram was developed for 2.5 h. Lane 1, WT; lane 2, C^468^->A; lane 3, C^516^->A; lane 4, C^674^->A; lane5 C^704^->A. The arrows indicate the location of the MMP-9 monomer (lower arrow) and MMP-9 homodimer (upper arrow).

The MMP-9 homodimer is reduction sensitive indicating at least one intermolecular sulfhydryl bond. Two additional non-conserved Cysteine residues, Cys^674^ and Cys^468^ are found in the carboxyl-end domain of the enzyme. RP-HPLC chromatography of the CNBr cleavage products from monomer and dimer with subsequent digestion with Trypsin and V8 proteases followed by amino acid residue sequencing allowed us to isolate two peptides from the monomer that were bridged by a sulphydryl bond in the cleavage products obtained from the dimer. Identification of these peptides suggested that dimerization of MMP-9 occurs due to the formation of an intermolecular disulfide bridge involving at least one of the three Cys residues (Cys^468^, Cys^516^ and Cys^704^) found in the carboxyl-end domain of MMP-9. Among these Cys^516^ and Cys^704^ are conserved and engaged in an intramolecular disulfide bridge as evident from the crystal structure of the homologous carboxyl end domain of MMP-2 [31], MMP-9 [39] and collagenase [40] leaving the Cys^468^ as a sole candidate residue involved in the dimer formation. To confirm this conclusion we analyzed the dimer forming ability of the MMP-9 mutants in which each of the three Cys residues was substituted for Ala or Gly. Each of the mutants was expressed in p2aHT2A cells and the secreted forms of MMP-9 were analyzed by zymography of the conditioned media. MMP-9 bearing a substitution of Ala for Cys^468^ was unable to form dimers (Fig. 6, lane 2) while mutants with substituted residues Cys^516^, Cys^674^, or Cys^704^ formed dimers as well as the wild type enzyme (Fig. 6, lane 3). These results demonstrate that the reduction sensitive homodimer of MMP-9 is held together by the intermolecular disulfide bridge between the non-conserved Cys^468^ residues positioned in the middle of a unique proline-rich region within the hinge domain.

**Figure 7:**
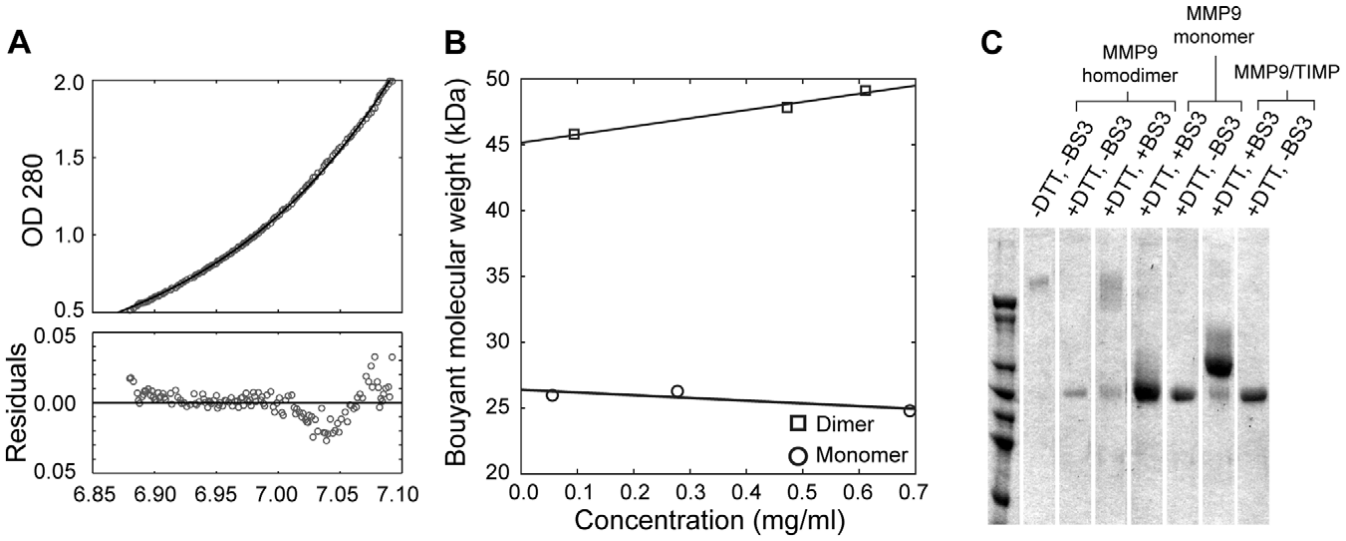
Sedimentation Equilibrium Analysis and BS3 crosslink of MMP-9 monomer and homodimer. Each molecular species was sedimented to equilibrium at 9000 rpm starting from 3 initial concentrations. Apparent buoyant molecular weights were obtained from non-linear least squares fits of the equation for equilibrium sedimentation of a single species to the corresponding concentration profiles (see methods). (**A**) The sedimentation profile (open circles) obtained for the MMP-9 monomer at its highest initial concentration shows the agreement with the non-linear least squares fit (solid line) which corresponded to an M_B,App_ of 24,800. The optical density range, 0.5 to 2.0, corresponds to a solute concentration range of 3 to 12 µM (0.28 to 1.1 mg/mL). (**B**) The apparent buoyant molecular weights (M_B,App_) of MMP-9 monomer (open circles) and homodimer (open squares) obtained as described above are plotted against the mid-point concentration of the corresponding sedimentation profile. (**C**) MMP-9 monomer and homodimer were reacted with the reduction insensitive cross-linking agent BS3 in solution (see Methods) and subjected to NaDodSO_4_ - PAGE (5%) analysis with or without post reaction DTT treatment. Molecular mass markers on the left correspond to 212 kDa, 170 kDa, 116 kDa, 94 kDa, 76 kDa, 67 kDa and 53 kDa.

The MMP-9 dimer was initially isolated as a secretion product of the cells in which MMP-9 was present in an excess relative to TIMP-1. Recently evidence has been presented that the recombinantly expressed hemopexin-like domain of MMP-9 can form spontaneous non-covalent homodimers in vitro [39]. These results suggest that pro-MMP-9 monomer might form spontaneous homodimers, an intriguing mechanism potentially regulating the amount of enzyme able to diffuse along the substrate surface. We have examined this possibility using purified protein in sedimentation equilibrium and cross-linking experiments (Fig. 7), and failed to observe evidence of spontaneous dimerization of the enzyme. Sedimentation equilibrium profiles for the purified monomer at three initial concentrations of solute show no significant deviation from the equation for a single sedimenting species even at the highest initial concentration, 0.7 mg/ml (Figure 7A, M_B, App_ = 24.8 kDa). The partial specific volume of the monomer has not been measured, but using an estimated value [41], 0.7328, gives an apparent molecular weight of 94.4 kDa, well within the range expected for the monomer. Moreover, the apparent buoyant molecular weights (M_B, App_) obtained at each initial concentration of solute were not significantly different (Fig. 7B). Like the monomer, the dimer sedimented as a single species with the M_B, App._ = 45 kDa determined by extrapolation to infinite dilution (Fig. 7B). The ratio of the extrapolated M_B, App._s of monomer to dimer is 1.731 where a value of 2.0 would be expected, suggesting that the dimer form has a partial specific volume about 4.5 percent larger than the free monomer since the monomer form obtained by reduction of the dimer has exactly the same electrophoretic mobility [67].

Thus, sedimentation analysis provided no evidence of spontaneous dimerization of MMP-9 in solution at concentrations comparable to those where most of the recombinant hemopexin-like domain of the enzyme was found in the dimer form [39]. Cross-linking experiments with MMP-9 monomer confirm this conclusion (Figure 7C). Cross-linking of MMP-9 monomer with BS_3_ (Figure 7C lane 4) failed to produce enzyme species with the MW of the MMP-9 dimer under conditions where the preexisting dimer (Figure 7C lane 3) or the non-covalent proMMP-9/TIMP-1 complex (Figure 7C lane 6) were crosslinked very efficiently. These results suggest that the C-terminal domain dimerization interface observed by Cha and co-workers [39] is hidden in the full length fully folded secreted enzyme.

### Inactivation of MT1-MMP Dependent Pericellular Collagenolysis Has a Detectable Negative Effect on the Force Development in Miniaturized 3D Tissue Constructs

The ability of membrane bound MMP complex to diffuse along a collagen fiber could potentially impact morphogenetic events involving cell-ECM binding and remodeling. To examine this possibility, we used 3D collagen based tissue constructs populated by embryonic fibroblasts in which cells develop contractile tension and compress and stiffen the collagen matrix. Measurement of the force vs. strain relationship characterizes the stiffness of these tissue constructs [42]–[44]. Importantly, the passive properties of the extracellular matrix can be separated from the active tension developed by cells by measuring constructs before and after the addition of cytochalasin D (an actin capping agent). The cellular and matrix contributions to the force are additive so that subtracting the passive (matrix) from the total curve yields the force-strain response of the cells (the “active” curve) [42], [45]–[47]. To make these measurements, we used a 3-D Tissue Stretching Robot [48] designed to perform simultaneous mechanical measurements on miniaturized fibroblast populated tissue matrices.

**Figure 8:**
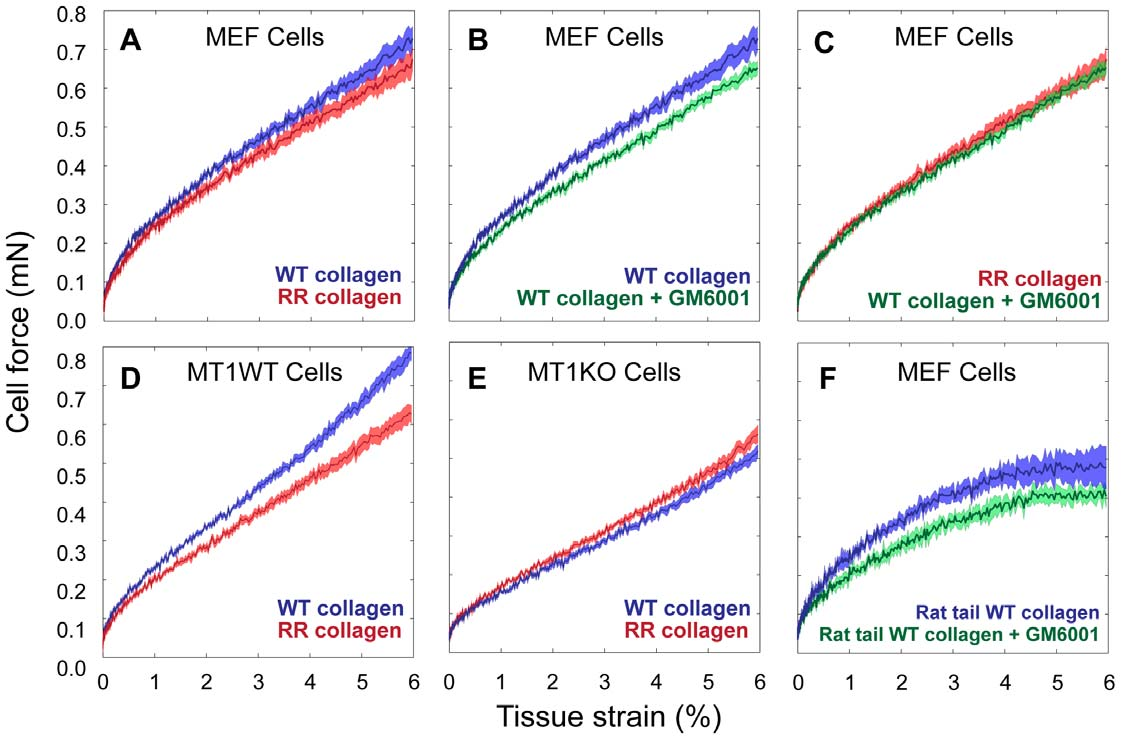
Effects of inhibition of pericellular collagenolysis on active cell forces (Fc) in stress-strain profiles of 3D tissue constructs. Embryonic fibroblasts from BALBC (MEF), MT1-MMP wild type (MT1-WT) or MT1-MMP knockout (MT1-KO) mice were cultured in either wild type (WT, black lines) or collagenase resistant (RR, red lines) mouse tail collagens. Each plot is the average of 6–8 tissue samples and the presented differences are highly significant (P<0.001). The results show a consistent reduction in force upon inhibition of collagenolysis either with MMP inhibitor Galardin (GM), use of collagenase resistant collagen or elimination of membrane tethered collagenase MT1-MMP (MT1-KO). Importantly, Galardin has no effect on the force when used to inhibit MMPs in MEF cells on the background of RR collagen (p = 0.78). **Fc = Ft–Fm**.

To assess the role of the pericellular collagenolysis on cell force, we utilized a combination of pharmaceutical and genetic means. We used GM6001, a broad spectrum MMP inhibitor, to assess the effect of inhibition of all or most MMP activities. Most importantly we used three types of collagen preparations and three types of mouse embryonic fibroblasts in permutations to pinpoint a more specific effect of pericellular collagenolysis and MT1-MMP in particular (Fig. 8). These include mouse embryonic fibroblasts from BALBC mice (MEFs), matched mouse embryonic fibroblasts from MT1-MMP wild type (MT1-WT) and MT1-MMP knockout (MT1-KO) mice [49], rat tail collagen, mouse tail collagen isolated from matched wild type (WT) and genetically modified mice with collagenase resistant collagen [50]. Inhibition of MMP activity with GM6001 at 25 µM final concentration resulted in a statistically significant (P<0.001) drop of cell force in tissue constructs utilizing MEF cells (panel B) or MT1-WT cells and either rat tail or WT mouse collagen (not shown). GM6001 did not have an effect on force in tissue constructs made from RR collagen (panel C). The force – strain plots for tissues populated with MEF cells on RR and WT mouse collagens plus GM6001 overlap, suggesting that the entire effect of the inhibitor is due to inhibition of collagenolysis. We next examined the effect of collagen digestion on cell force in tissue constructs from MEF cells embedded in mouse tail WT and collagenase resistant collagen (RR, panel A). The cell force drop (p<0.0001) was observed in every instance where collagenolysis was negatively affected by either MT1-MMP knockout or RR mutation in the resistant collagen. The reduction in force is visible from very small strains (<0.5%) and increases through the range tested resulting in a maximal difference of 0.04 mN at 6% strain. These results demonstrate that peri-cellular collagenolysis contributes to the force cells can exert on the collagen matrix in 3D cultures.

## Discussion

The restricted mobility of the ECM macromolecules and the cell surface-tethered MMPs raises the question: how do these collagen modifying proteases navigate the congested space of the ECM to acquire their specific cleavage sites? Our previous experiments [12], [13] along with the results presented here firmly establish that MMPs utilize a remarkable surface diffusion mechanism for substrate interaction. MT1-MMP, MMP-1, -2 and -9 can diffuse laterally on the collagen substrate surface without noticeable dissociation. Both MMP-2 and -9 exhibit random diffusion on the surface of the fibrils. Most instructive is the fact that the rate of diffusion of MMP-2, and -9 is not substantially affected by complex formation with inhibitors TIMP-2 and -1 respectively. The C-terminal domain of MMP-2 is required for diffusion on the surface of gelatin and presumably comes in contact with the substrate. A large portion of the C-terminal domain's solvent exposed surface is occupied by the bound inhibitor when the two are in complex [33]. The inhibitor must, therefore, be strategically positioned on the surface of the C-terminal domain not to interfere with the diffusion process. By contrast, the dimerization of the MMP-9 renders the enzyme immobile on the surface of gelatin layer as well as the collagen fibril. While this manuscript was in preparation, Rosenblum et. al. using Atomic Force Microscopy observed lateral motion of single molecules of MMP-9 on the 3/4 fragment of a triple helical collagen produced by MMP-8 digestion of collagen-II [15]. Initial binding of the protease was followed by diffusion along the fragment with accumulation at the carboxyl terminus of the cleaved collagen.

**Figure 9:**
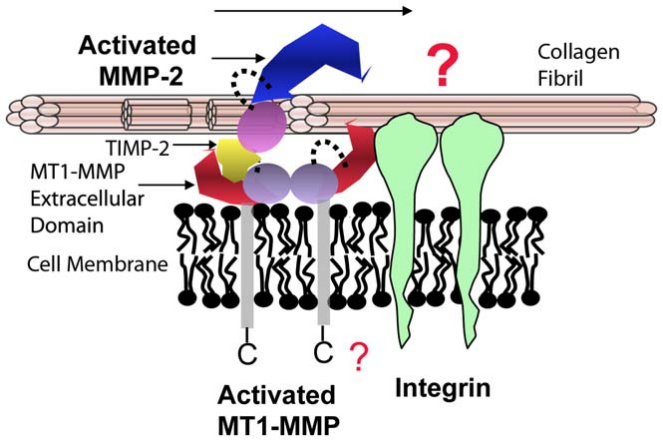
The Mobile Cell Surface - Collagen Substrata Collagenolytic Interface. All components of MT1-MMP cell surface collagenolytic complex are shown to be mobile on the collagen fibril. Activated MT1-MMP can initiate the digestion of collagen fibril and is a Brownian Ratchet capable of biased diffusion similar to the secreted collagenase MMP-1. MMP-2/TIMP-2 complex is capable of random lateral diffusion on the same substrate and can assist in MT1-MMP dependent degradation of the fibril to small peptides. Integrin, a collagen receptor, can bind to collagen fibril and potentially interact with the MT1-MMP complex. The exact mechanism of that interaction remains to be elucidated. The arrow indicates hypothetical directionality of transport.

Most interestingly, compared to the random diffusion exhibited by MMP-2 and -9, we find that the motion of the activated collagenolytic extracellular portion of the membrane protease MT1-MMP is dominated by biased diffusion similar to MMP-1 [13]. The mechanism of the biased component of the motion is accurately described by the Burnt Bridge Brownian Ratchet model [13], [14] driven by collagen proteolysis, not ATP hydrolysis. Together these findings suggest that the entire cell surface collagenolytic complex of (MT1-MMP)_2_/TIMP-2/MMP-2 responsible for cell surface activation of MMP-2 is mobile relative to the underlying collagen substrata and comprises an essential part of a mobile cell surface - ECM interface (Fig. 9). The presence of integrin promotes the second autocatalytic step in MMP-2 activation ((Goldberg, unpublished data), [51]) suggesting a functional interaction and close proximity between these two (Fig. 9). These findings have profound implications for mechanistic understanding of the role of MMPs in spatially controlled peri-cellular collagenolysis, cell spreading, motility and invasiveness.

The collagen fibrils consist of polymerized triple helical collagen monomers tightly packed in a quasi-helical lattice [52]. Each collagen monomer contains a single MMP-1 collagenase cleavage site. These sites are lined up along the fibril axis at 67 nm intervals and so are digested in a processive manner by the MT1-MMP (or MMP-1) as it undergoes biased one dimensional diffusion along the fibril. The cleavage of three collagen chains in a triple helical monomer by the collagenase creates a “burnt bridge” [53] which prevents the backward diffusion of the enzyme thus rectifying the random diffusive motion [13] of the enzyme into a transport mechanism.

The intact collagen fibril is not a substrate for MMP-2. After the initial cleavage of the collagen fibril by MT1-MMP the presence of activated MMP-2 [10], [17] in the cell surface complex renders it capable of complete digestion of the collagen fibril to small peptides. The denaturation temperature of the collagen monomers of most mammals is, surprisingly, slightly lower than their physiological temperature [38], [54]. The triple helical denaturation process is slow enough at physiologic temperatures to allow for the incorporation of the collagen monomers into fibrils before monomer denaturation can occur. Once fibrils are formed, the constituent monomers are stabilized by the thermodynamic favorability (negative free energy) of the fibril formation [55]. Cleavage of the monomer by MT1-MMP further lowers its melting point allowing it to unfold, making possible the digestion of the monomer by the gelatinolytic activity of MMP-2.

What is the structural basis for MMP random processive diffusion? Since substrate surface diffusion was proposed by Adam and Delbruck [56] many enzyme systems have been found to utilize processive diffusion for substrate interaction including restriction endonucleases [57]–[59], DNA regulatory proteins [60]–[62], cellulases [63] and now MMPs. One class of these processive enzymes have structures which permit them to partially wrap around their extended, rod like polymer substrates [64] and are characterized by a groove or saddle shape allowing for an extended surface interaction with the substrate permiting them to slide on the surfaces of their polymer substrates. For example, the DNA binding sites of restriction endonucleases EcoRV [57] and BamHI [58], and the lac repressor [65] consist of a groove formed by the cooperative action of each member of the non-covalent homodimer that constitutes the active form of these proteins. Considerable flexibility between members of the dimer is important in going from the free to the bound state [58], [65]. There is evidence that the MMPs belong to this class of protein.

The structures of MMP-1, -2 and -9 consist of two domains connected by a linker region: an approximately globular catalytic domain and an approximately cylindrical, carboxyl terminal, hemopexin domain, [27], [31], [66]–[68]. Thus, while either domain alone does not permit an extended interaction surface with the rod-like collagen substrate, together they would permit the formation of such a structure involving the catalytic domain and blade II of the hemopexin domain [69]. Our previous data support this type of mechanism of diffusion. The C-terminal domain of MMP-2 [12] is essential for the diffusion of MMP-2 on gelatin coated surfaces despite the fact that the catalytic domain alone will bind to the substrate. In the case of MMP-1, digestion of native collagen fibrils, which occurs by random processive diffusion [13], also requires the intact carboxyl terminal domain [70], [71]. Computer simulations show that the formation of such a substrate interface by MMP-2 requires inter domain flexibility [69]. Recently, conformational flexibility of both MMP-1 and monomeric MMP-9 conferred by the linker region has been demonstrated directly [41], [72], [73]. The flexibility of other MMPs is widely assumed based on the presence of the unstructured linker domain.

The TIMP inhibitors must, therefore, be positioned on the C-terminal domain not to interfere with any potential collagen/gelatin interaction surface formed cooperatively by the two domains. The interaction between TIMP-2 and the carboxyl terminal domain of MMP-2 has been described [33]. TIMP-2 engages the carboxyl terminal domain of MMP-2 on the sides of blades III and IV of the hemopexin ß-propeller of MMP-2 leaving blades I and II free. Thus, bound TIMP-2 (about the same thickness as the ß-propeller) points away from the axis of the propeller on the side farthest away from the NT domain. TIMP-2 binding should not interfere with any possible extended interaction surface formed by adjacent regions of the NT and carboxyl terminal domains [69]. A crystallographic study of the carboxyl terminal domain of MMP-9 [39] suggests that its TIMP1 binding site is also on blades III and IV of the ß-propeller and thus a similar argument may apply to MMP-9/TIMP-1. In consequence of these observations, we hypothesize (i) that the ability of the MMPs to engage in random processive diffusion is based on an extended substrate interaction surface formed by the cooperative action of the two domains. And (ii) conformational freedom of the two domains is essential to the diffusion process.

The observation that the MMP-9 dimer is immobile on collagen fibrils is of significance in understanding the mechanisms of MMP-9 interaction with collagen fibrils. We have demonstrated that the reduction-sensitive dimer contains an intermolecular disulfide bridge between the Cys^468^ residues which lie in the linker region of the monomer. This conclusion is based on the peptide mapping of the purified monomer and dimer and the inability of an MMP-9 point mutant (Cys^468^->Ala^468^) to form dimers when expressed in mammalian cells. The failure to observe spontaneous dimer formation by monomers using analytical ultra centrifugation and crosslinking experiments suggests that the dimerization occurs intra-cellularly although the non-covalent dimer formation of the recombinant hemopexin domain has been observed in solution [39]. These observations suggest that the formation of the enzyme dimer by a Cys^468^ mutant of recombinantly expressed MMP-9 [74] is due, most likely, to the not entirely faithful folding of the enzyme in that system.

What consequent structural changes could render the homodimer immobile on the collagen and gelatin substrate surfaces? First, the MMP-9 homodimer contains two fibronectin-like gelatin/collagen binding domains. If both domains can engage in gelatin or collagen binding simultaneously, a higher binding affinity could result which might limit site to site mobility or diffusion. A ten fold higher affinity of the dimer for gelatin has in fact been noted (Collier and Goldberg unpublished data). Second, the covalent linkage between the constituent monomers that is responsible for the dimer is a constraint upon the relative motion of the catalytic and C-terminal domains not obtaining in the monomer. Steric hindrance resulting from this constraint might prevent the formation of any cooperative interaction between the domains necessary for diffusion. Our sedimentation equilibrium results indicate that the dimer form of the MMP-9 has a higher partial specific volume than purified monomer suggesting a more open, less dense conformation which might be a result of such constraints.

In addition to structure of the MMPs that allows the enzyme to diffuse on the substrate surface the recently proposed model of collagen proteolysis by Perumal et all suggests that the structural features of the substrate can explain the biased component of the motion [16]. In this model after initial recognition and cleavage the sequence in which consequent MMP-1 cleavage sites are exposed is determined by the process of fibril depolymerization and the interaction of the enzyme with α2 chain that allows access to sites N-terminal to the initial cleavage. Thus the process of collagenolysis would proceeds more rapidly in N- to C-terminal direction accompanied by a very slow process of lateral movement.

It is increasingly clear that the spatial control of ECM proteolysis by MMPs involves the sequestering of secreted MMPs to specific structures on the cell surface and ECM while processive diffusion on the substrate surfaces, has emerged as the strategy that MMPs deploy in modifying cell surface/ECM interface.

Collagen degradation in vitro by either MT1-MMP or MMP-1 involves a biased diffusion of the enzyme on the fibril surface where the mechanism of the transport is a “burnt bridge” Brownian Ratchet [53] which requires the proteolytic activity of the enzyme. If the energy responsible for the biased motion of the MT1-MMP comes merely from restricting the diffusion of the enzyme to only one side of the cleaved collagen monomer helix, the result is a “burnt bridge” Brownian ratchet which generates a relatively small stall force of approximately 0.1 pN. (The energy driving the motion is only k_B_T.) We have described a theoretical model [14] in which proteolysis and fibril unpacking yield far more energy than that required merely to restrict diffusion to one side of the cleavage. There are two mechanisms by which a larger force might be generated. The large energy due to proteolysis and fibril unpacking could be coupled to the motion of the MT1-MMP over a portion of its travel along the collagen fibril. In such a case the stall force increases, but only several fold because it is still dominated by the simple diffusion of a Brownian ratchet over the remainder of its travel.

An alternative is based on the cell surface dimerization of MT1-MMP [21], [22], which theoretically could provide a continuous large driving force experienced by first one and then the other member of the dimer over the entire range of its motion. An MT1-MMP dimer moving on adjacent monomer helices of the fibril could produce a stall force approaching 4.5 pN, a magnitude comparable to that generated by ATP driven motors. Structural, mutagenesis and domain replacement studies indicate that both the C-terminal and transmembrane domains are involved in MT1-MMP dimerization on the cell surface that is important for cell surface activation of MMP-2 proenzyme and the cell surface collagenolytic activity associated with cell migration and invasion [75], [76]. It is important to note that the cell surface collagenolysis being discussed here is detected by bio-assay: plating cells transfected with MT1-MMP constructs on collagen layers and observing cell migration and the appearance of zones of degraded collagen, or by observing the penetration of transfected cells into preformed collagen gels. Nothing in the reported experiments would be inconsistent with MT1-MMP monomers having the ability to diffuse and cleave collagen fibrils. The elution profile of the refolded recombinant MT1-MMP from size exclusion chromatography on Superdex 200 10/300 GL (data not shown) indicates that MT1-MMP used in this study is a monomer in solution. Whether this monomer can dimerize in the presence of the substrate is not known and requires further investigation.

Although there is no experimental evidence available to support either of the models of force generation the above considerations motivated us to examine whether the activity of MT1-MMP has any effect on the force exerted by cells in 3D collagen constracts.

Our results show that MT1-MMP activity contributes to the contraction forces developed by fibroblasts populating 3D collagen tissue constructs. The cells develop contractile tension and compress and stiffen the collagen matrix. The force vs. strain relationship characterizes the stiffness of these tissue constructs [77]–[79]. To a first approximation, the tissue stress is the sum of matrix and cell stress [45]–[47] so that subtracting the matrix contribution (determined in the presence of CD) from the total yields the cellular stress. In every case where the activity of MT1-MMP was attenuated, whether by genetic ablation, chemical inhibition or matrices formed with collagen lacking the enzyme cleavage site, the cellular force developed was significantly reduced. At maximum, the reduction was 0.04 mN (at 6% strain) representing about 4% of the total cellular force. This result, although very intriguing, raises more questions than provides answers.

Are these calculated magnitudes consistent with the MT1-MMP dependent force demonstrated in the tissue constructs? Each tissue construct is seeded with 5×10^5^ fibroblasts. Thus, the forces cited above, would require approximately several hundred force producing contacts per cell. Although the number of integrin dependent cell-matrix contacts has not been measured, visual inspection of micrographs of 3-D collagen matrix-imbedded fibroblasts [80], [81] suggest that numbers in low 100 s are not unreasonable. Thus, direct force generation by MT1-MMP is biophysically feasible.

Alternatively, the contribution to cell force can be indirect with MT1-MMP contributing to the organization of the interaction of the collagen fibril with the membrane complex involved in cell locomotion. Thus the MT1-MMP could influence force development through an effect on the organization of the cell-matrix attachments as they are remodeled during tissue construct formation. Cells connect to the ECM via structures that bind to actin filaments, e.g., integrins and cell migration involves actomyosin contraction that generates traction forces [82]. Since MT1-MMP is likely to be associated with integrin on the cell surface it may well play a role in deploying cell-matrix attachments and thus contribute to the magnitude of force generated by affecting the number and quality of matrix attachment sites. The effect of varying attachment sites on force generation has been noted. In 2-D substrates, for example, cell migration involves traction forces on the substratum through the actin cytoskeleton and focal adhesions plaques (FAs) [83]. In relatively stationary cells, there is a positive correlation between the traction force and the size of the FAs [84], [85]. The differences in the traction force–FA size relationship under these conditions may be related to the number of FAs and how these FAs link to actin filaments. Furthermore, the effect of attachment site density on force production is demonstrated by studies which show that the density of ECM proteins controls the level of integrin-ECM adhesive interaction and modulates cell migration speed. At low adhesiveness, the cell cannot form strong and stable adhesions at the front to generate traction force; at high adhesiveness, the cell cannot break the cell-ECM adhesions at the rear. On ECM proteins with a density gradient, cell migration is driven by haptotaxis, e.g., from the low-density, less adherent area to the high-density, more adherent area [86].

The experiments presented here do not distinguish between the direct and indirect mechanism by which directional collagen degradation contributes to cell stress because in all cases the inhibition of MT1-MMP is present during construct development and remodeling as well as during the experimental force measurement. A direct effect on cell force production would be defined operationally as an acute inhibition with the inhibitor of protease activity applied just prior to the experimental force measurement. This could be done with stress relaxation experiments [42], [44] on FPMs with normal collagen matrices populated by MT1-MMP wild type cells. Any drop in force caused by the application MT1-MMP inhibitors to stretch pre-conditioned FPMs held at a steady level of stress and strain would indicate the magnitude of a direct contribution to cell force by MT1-MMP. Our attempts at this kind of experiment did not yield a reliable result. Nevertheless the contribution of cell surface proteolytic activity to the cell force generation in 3D constructs is an intriguing result opening new avenues for further investigations.

## Materials and Methods

### Enzyme Purification and Labeling

Cultured Human cells were grown in RPMI 1640 supplemented with 2 mM Glutamine and 5% FCS. MMP-2 pro-enzyme as well as MMP-2 pro-enzyme-TIMP-2 complex were purified from conditioned medium of p2aHT7211A cells [10], [17]. MMP-9 pro-enzyme, MMP-9 pro-enzyme -TIMP-1 complex, and MMP-9 pro-enzyme homo-dimer were purified from conditioned medium of p2a92c8 cells [30].

Purified enzymes were labeled with the fluorescent dye Alexa Fluor™ 488 for fluorescence photo bleaching recovery (FPR) and fluorescence correlation spectroscopy (FCS) experiments as described previously [12].

### Protein expression and purification

Following the successful cloning and sequence verification, MT1-MMP construct was transformed into BL21-CodonPlus (DE3)-RIL *E.Coli* competent cells (Stratagene) under Amp selection in LB media. Protein expression was induced when bacteria reached an optical density of between 0.8 and 1.0 using 1 mM IPTG. After induction for 4 hours, bacteria were centrifuged at 8000RCF in a JLA 8.1000 rotor at 4C for 20 minutes. The supernatant was discarded and cells re-suspended in solution buffer (50 mM Tris-HCL pH 8.0, 25% Sucrose, 1 mM EDTA, 0.01% Sodium azide, 1.54 mg/ml DTT). Following their resuspension, an equal volume of lysis buffer (50 mM Tris-HCL pH 8.0, 1% Triton X-100, 1% Sodium deoxycholate, 100 mM NaCl, 0.01% Sodium Azide, 1.54 mg/ml DTT) was added, followed by 800 ml of 50 mg/ml lysozyme and 1 ml of 4 mg/ml DNAse I. The solution was then incubated at room temperature for 1 hour while stirring, and then sonicated until no large cellular debris could be seen by eye. The lysed cells were then centrifuged at 6000 RCF for 15 min in a JLA 16.250? at 4C. The supernatant was again discarded, leaving a relatively impure inclusion body. The inclusion body was washed and pelleted at 6000 RCF three times using a wash buffer containing Triton (50 mM Tris-HCL pH 8.0, 0.5% Triton X-100, 100 mM NaCl, 1 mM EDTA, 0.01% sodium azide, 0.154 mg/ml DTT). A sonicator was used to resuspend the inclusion body after each wash step. The final wash step (50 mM Tris-HCL pH 8.0, 100 mM NaCl, 1 mM EDTA, 0.01% sodium azide, 0.154 mg/ml DTT) removes most of the detergent from the inclusion bodies. The final, purified inclusion body was dissolved into a minimal volume of 6 M guanidine hydrochloride (GuHCL), 10 mM Tris pH 8.5, 10 mM BME. Solubilized MT1-MMP inclusion bodies were then aliquotted into 1 ml fractions and frozen at −80C for storage.

### MT1-MMP Refolding

The protein was refolded using the Arginine oxidative refolding method. Briefly, a 400 ml volume of Arginine refolding buffer (400 mM L-Arginine, 100 mM Tris pH 8.5, 2 mM CaCl2, 5 mM reduced glutathione, 0.5 mM oxidized glutathione, 0.2 mM PMSF) was prepared and chilled. Into this buffer, three injections of 500 ml of solubilized inclusion body were made over the course of 1 hour (0, 30 min, 60 min). The resulting solution was stirred slowly overnight at 4C. The following day, the protein was removed by filtration, concentrated using YM-30 (30 kD cutoff) filter membrane (Millipore) to a volume of 2 ml and purified using size exclusion chromatography using High Load 16/60 Superdex S200 prep grade columns (GE Healthcare). The protein eluted at the expected volume for its predicted size.

### FPR measurement and Analysis

FPR of fluorescent labeled purified enzymes bound to gelatin layers and subsequent analysis were performed and analyzed as described previously [12] with the exceptions that coverslips were coated with gelatin by a 40 µg/ml (gelatin) covering solution and the FPR buffer was modified by the addition of 2.5 mM Trolox to inhibit photobleaching making the buffer consistent with that used for FCS [24].

### FCS measurement and Analysis

Two-photon excitation FCS was performed on fluorescent labeled, purified enzymes bound to collagen fibrils in hydrated collagen gels and experimental FCS curves were obtained as described for MMP-1 [13]. Briefly, a selected individual fibril was centered under the 5-mW laser beam and illuminated for 90 to 120 s to achieve a steady state fluorescence. Primary fluorescence data was then immediately collected for 300 s with a bin time of 400 µs. The 400 µs record is dominated by shot noise; however, increasing the bin time to 40 ms reveals spikes of fluorescence attributable to single fluorescent molecules passing through the laser beam. A spatial backround filter determines the number and position of the spikes and processes the 400 µs primary record to remove the background noise around the spikes. The processed 400 µs record is passed to a software correlator which produces the experimental FCS curve.

The expected FCS function, G(τ), arising from one dimensional diffusion of the labeled molecule is:
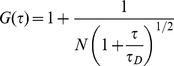
(1)where 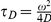, ω is the beam waist of the focused laser beam, N is the average number of fluorescent particles in the laser-illuminated area, and D is the diffusion coefficient of the diffusing species. Experimental correlation functions for MMP-2 and -9 bound to collagen fibrils showed a consistent, small deviation from equation 1.0 which proved to be due to photo bleaching of the Alexa 488 label. Equation 1.0 may be modified ([87]–[89]) to account for fluorophore photo-bleaching:
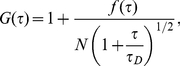
(2)where the function *f(τ)* is defined as
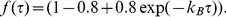
(2a.)The parameter *k_B_* is the photo-bleaching rate constant, and 0.8 is the apparent fraction of molecules susceptible to photo-bleaching. The value 0.8 is consistent with previous practice [87] and our own preliminary analysis of experimental FCS curves. When equation 2 was fitted to the experimental correlation functions for MMP-2 and -9, the inconsistency was no longer apparent with *k_B_* and D both free parameters. A set of FCS data for MMP-9 (four trials) was collected for reference at 5 mW of laser power. Equation 2 was fitted to each of the obtained FCS curves yielding four values of the photo-bleaching rate constant *k_B_* for which a mean value and standard deviation of 1.8±0.47 s^−1^, respectively, were determined. To demonstrate that the deviation from equation 1 was due to photo-bleaching of the dye, the photo bleaching rate constant for collagen-fibril bound MMP-9 was determined directly and the result compared to the value cited above. The fluorescence intensity from a pre-positioned fibril was measured at 400 µs intervals after switching on the 5 mW laser beam. Equation 2a was fitted to the first 0.5 seconds of the fluorescence decay yielding a value for the photo-bleaching rate constant *k_B_*. A mean value and standard deviation of 2.3±0.19 s^−1^, respectively, were determined from five individual measurements. The values of *k_B_* obtained by the two different methods are not significantly different (at a P-value of 0.06 for Welch's approximate t-test). Thus the deviation of the experimental correlation functions from ideal one-dimensional diffusion is quantitatively accounted for by the observed amount of photo bleaching.

An identical direct photo bleaching experiment was performed for collagen-fibril bound, Alexa 488- labeled MT-1MMP which yielded a mean *k_B_* and standard deviation of 2.6±0.4 s^−1^, respectively, for this enzyme. MT1-MMP may be expected to demonstrate motor (directional flow) activity based on the fact that it demonstrates collagenolytic activity. The expected correlation function arising from one dimensional diffusion and directional flow [23], corrected for photo-bleaching of fluorescently labeled MT1-MMP is consequently:
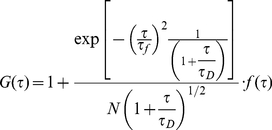
(3)where
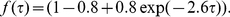
(3a.)Equation 3a is obtained by assigning the predetermined value 2.6 s^−1^ to the photobleaching rate constant.

Equations 1 through 3, as appropriate, were fitted to experimentally obtained FCS curves by two or three parameter, unweighted, non-linear least squares fitting using the Levenberg-Marquardt algorithm.

### Sedimentation, Cross-linking and SDS PAGE analysis

Analytical sedimentation was performed in a Beckman Optima XL-A ultracentrifuge at 20C in accordance with the procedures of the Beckman Manual. Solutions of MMP-9 monomer and homodimer were prepared in 25 mM Hepes (pH 7) buffer with 150 mM NaCl and 1 mM CaCl_2_. These were loaded into cells of an An50TI rotor and centrifuged at 12000 rpm. Sedimentation profiles on the XL-A ultracentrifuge were obtained by automatic scans of the UV absorbance (280 nm) of the cell at increments (approximately 0.01 cm) along direction of the gravimetric field. Sedimentation proceeded until three successive scans, each an hour apart, indicated no significant change in the sedimentation profile (about 12 hrs total sedimentation time). The buoyant molecular weights, M_B, App_, were obtained from two parameter, unweighted non-linear least squares fits of the sedimentation equilibrium equation [90] to the equilibrium profiles. SDS polyacrylamide gel electrophoresis (SDS-PAGE) was performed as described [27].

Cross-linking reactions were performed with or without BS_3_ at a concentration of 5 mM in 20 mM HEPES buffer supplemented with 150 mM NaCl and 5 mMCaCl_.2_. Reactions were performed on 3.8 pmol of MMP-9 monomer and MMP-9 monomer/TIMP complex or 1.35 pmol of homodimer enzyme in 10 µl reaction volume for 1 hour at 37°C and quenched by the addition of 5 µL SDS-PAGE gel loading buffer with or without DTT as indicated. Reactions were subjected to SDS - PAGE (5%) described above.

### Measurement of Force in 3-Dimensional Tissue Constructs

3D collagen based tissue constructs populated by chicken embryonic fibroblasts in which cells develop contractile tension and compress and stiffen the collagen matrix were constructed as described previously [42]–[44]. Miniaturized fibroblasts populated tissue rings (FPMs) were formed in a Teflon mold in a temperature-controlled environment. We used a 3-D Tissue Stretching Robot [48] designed to perform simultaneous mechanical measurements on 32 FPMs.

Briefly, two-axis robotic control was achieved through a combination of stepper motors and linear actuators to bring a cantilever type isometric force transducer into contact with each sample. The head of the transducer consisted of a 1 mm diameter stainless steel dowel pin with an “L” shaped bend approximately 6 mm from the tip. The horizontal portion of the tip was positioned above the center of each FPM such that its central axis was parallel to those of the two supporting anchors in the culture well. The dynamic stiffness and tension of the FPM were measured at various strain levels by lowering the probe tip and stretching the tissue by depressing it along its central line of symmetry. The measured upward force vector was proportional to the membrane tension according to the ratio 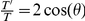 where T' is the measured upward force vector, T is the membrane tension and 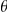 is the angle between the probe head and the tissue surface.

Mechanical variability, as well as slight differences in tissue thickness, resulted in a range of indentation depths from .7 and .8 mm which corresponded to a maximal tissue strain of approximately 6%–8%. The total time period for each stretch (i.e. indentation and relaxation) was 6.5 seconds. Each tissue sample was subjected to 3 identical stretches with a rest period of approximately 5 minutes in between each stretch. Following previously established methodology [91], the first stretch was considered a pre-conditioning stretch; the second and third stretches were averaged to produce the stress-strain profile for each sample. The relative contributions of cell and matrix (ECM) forces were separated from the initial stress-strain profile by incubating each FPM with 2 µM Cytochalasin D (CD) for 2 hours at 37°C in 5% CO_2_ immediately following the initial measurements. After this incubation period, samples were measured in an identical manner as before resulting in a stress-strain curve due solely to the ECM without active cellular contributions. Cell force was then determined as the difference between the pre CD (combined) profile and the post CD (matrix) profile. Measurements of cell force under each condition resulted in similarly shaped plots, which increased approximately linearly, from 1%–6% strain with a maximum force on the order of 1 mN.
